# Cell Surface Expression Level Variation between Two Common Human Leukocyte Antigen Alleles, HLA-A2 and HLA-B8, Is Dependent on the Structure of the C Terminal Part of the Alpha 2 and the Alpha 3 Domains

**DOI:** 10.1371/journal.pone.0135385

**Published:** 2015-08-10

**Authors:** Christoffer Dellgren, Jan O. Nehlin, Torben Barington

**Affiliations:** Department of Clinical Immunology, Odense University Hospital, Odense, Denmark; Massachusetts General Hospital, UNITED STATES

## Abstract

Constitutive cell surface expression of Human Leukocyte Antigen (HLA) class I antigens vary extremely from tissue to tissue and individual antigens may differ widely in expression levels. Down-regulation of class I expression is a known immune evasive mechanism used by cancer cells and viruses. Moreover, recent observations suggest that even minor differences in expression levels may influence the course of viral infections and the frequency of complications to stem cell transplantation. We have shown that some human multipotent stem cells have high expression of HLA-A while HLA-B is only weakly expressed, and demonstrate here that this is also the case for the human embryonic kidney cell line HEK293T. Using quantitative flow cytometry and quantitative polymerase chain reaction we found expression levels of endogenous HLA-A3 (median 71,204 molecules per cell) 9.2-fold higher than the expression of-B7 (P = 0.002). Transfection experiments with full-length HLA-A2 and -B8 encoding plasmids confirmed this (54,031 molecules per cell vs. 2,466, respectively, P = 0.001) independently of transcript levels suggesting a post-transcriptional regulation. Using chimeric constructs we found that the cytoplasmic tail and the transmembrane region had no impact on the differential cell surface expression. In contrast, ~65% of the difference could be mapped to the six C-terminal amino acids of the alpha 2 domain and the alpha 3 domain (amino acids 176–284), i.e. amino acids not previously shown to be of importance for differential expression levels of HLA class I molecules. We suggest that the differential cell surface expression of two common HLA-A and–B alleles is regulated by a post-translational mechanism that may involve hitherto unrecognized molecules.

## Introduction

The classical Human Leukocyte Antigen (HLA) class I molecules: HLA-A,-B, and -C bind and present intracellularly produced peptides on the surface of a wide variety of cells. The peptides may originate from the cell’s own proteome or from an intracellular pathogen, e.g. a virus. Once on the cell surface, the HLA-peptide complex is monitored by specific Cluster of Differentiation (CD8)+ cytotoxic T lymphocytes that recognize foreign peptides and kill the infected cells that present them by inducing apoptosis. Cancer cells can also be identified and terminated because of the mutated or aberrantly-expressed peptides they may present. HLA class I molecules consist of an extremely polymorphic transmembrane heavy chain forming the peptide-binding groove and a non-covalently associated beta-2-microglobulin (B2M). Different alleles bind different sets of peptides and certain alleles may influence the course of specific infections. For example, HLA-B*57:01 and HLA-B*27:05 are associated with slow progression of HIV infection while HLA-B*35:03 is associated with rapid progression [1–5].

Besides the qualitative differences, quantitative differences in expression levels are also of clinical importance. Reduced HLA expression is, indeed, a common evasive mechanism of intracellular pathogens and cancer cells leading to immune escape [6–9]. Moreover, recent data suggest that minor differences (up to three-fold) in the normal cell surface expression of the various alleles may be of importance for immune responses. Thus, Apps and colleagues found a correlation between the normal cell surface expression levels of HLA-C on CD3+ cells and progression of HIV infection [10]. Also, the incidence of severe graft-versus-host disease and non-relapse mortality in HLA-C mismatched allogeneic bone-marrow transplantation correlates with the expression level of the mismatched patient HLA allele on CD3+ cells [11].

Whereas HLA-A,-B, and -C are constitutively co-expressed on leukocytes, other cell types like striated muscle cells, hepatocytes and adult neurons completely lack expression in the absence of inflammatory signals [12, 13]. Moreover, we have recently found that several cell types in the body vary widely in the expression of the individual antigens. Thus, we found that expression of HLA-B was often low or absent on many types of human multipotent stem cells and on some differentiated cell types, while HLA-A expression was high on most cells [14, 15]. In mesenchymal stem cells, we found a 17- to 40-fold lower expression of HLA-B when compared to HLA-A [14]. These differences clearly exceed those found between HLA-C alleles in CD3+ cells and may have important implications for the immune responses. They are not caused by inhibition of transcription as the mRNA levels of HLA-A,-B, and -C were comparable [14]. In most cells, HLA-B expression could be induced by stimulation with Interferon γ (IFN-γ) to cell surface levels comparable to that of HLA-A. The mechanism that gives rise to markedly different constitutive expression of HLA-A and -B on the cell surface still remains to be elucidated.

Here we demonstrate that the differential constitutive cell surface-expression of HLA-A2 and -B8 is primarily determined by the coding sequences and therefore is most likely related to structural differences between these homologous molecules. We have determined which part of the HLA-B8 coding sequence is important for the impaired expression relative to HLA-A2 by using chimeric constructs where the effect of different parts of the molecules was analyzed by transfecting cells and measuring the cell surface expression. We found that especially the alpha 3 domain is important for the differential expression of HLA-A2 and -B8. Our data suggest that a hitherto unrecognized posttranslational mechanism regulates the expression of HLA and may control the variable constitutive expression of different HLA alleles in different tissues.

## Materials and Methods

### Plasmids

Full length coding sequence of HLA-A*02:01:01 was Polymerase Chain Reaction (PCR) amplified (forward primer: ATGGCCGTCATGGCGC, reverse primer: TCACACTTTACAAGCTGTGAGAG) using cDNA from a telomerase-immortalized mesenchymal stem cell line hMSC-Tert4 [16] as template. Full length coding sequence of HLA-B*08:01:01 was PCR amplified (forward primer: ATGCTGGTCATGGCGC, reverse primer: TCAAGCTGTGAGAGACACATCA) using cDNA from adipose-derived MSC-661 cells [14] as template. Total RNA purification and cDNA synthesis were performed as described below. Amplified PCR products were cloned into the mammalian pEF6/V5-His TOPO TA expression vector (Life technologies), and verified using BigDye terminator V3.1 (Life technologies) sequencing technology.

### Chimeric constructs

HLA-A2/-B8 chimeric constructs were generated by PCR amplifying (see RNA isolation and RT-PCR section) the two fragments that were to be fused using a reverse primer for the leading fragment that was complementary to the forward primer of the trailing fragment. The two fragments were fused in a second PCR, and the amplified product cloned into the mammalian pEF6/V5-His TOPO TA expression vector, and verified using BigDye terminator V3.1 (Life technologies) sequencing technology. The forward primer used for leading HLA-A*02 fragments was: ATGGCCGTCATGGCGC, and for leading HLA-B*08 fragments: ATGCTGGTCATGGCGC. The reverse primers used for the leading fragments were (in the order of the peptides they encode): A2_1-176_ and B8_1-176_: TCCAGGTAGGCTCT, A2_1-308_ and B8_1-308_: ATGCCCACGATGGGGA, A2_1-284_ and B8_1-284_: ATCACAGCAGCGACCACA. The reverse primers used for trailing A2 and B8 fragments were: TCACACTTTACAAGCTGTGAGAG, and TCAAGCTGTGAGAGACACATCA, respectively. The forward primer used for the trailing fragments were: A2_177-341_ and B8_177-338_: AGAGCCTACCTGGA, A2_309-341_ and B8_309-338_: TCCCCATCGTGGGCAT, A2_285-341_ and B8_285-338_: TGTGGTCGCTGCTGTGAT. Gene construct analysis was facilitated by the use of the CLC Genomics workbench software (CLC bio, Qiagen).

### Cell culture and HLA typing

The HEK293T cell line [17] was grown in Dulbecco's modified eagle's medium with L-glutamine (Life Technologies) supplemented with 10% (v/v) fetal bovine serum (Life Technologies), 100 μg/ml streptomycin and 100 U/ml penicillin (Life Technologies).

Genomic DNA was purified from HEK293T with the QiaAmp DNA Mini-kit (Qiagen). A low-resolution HLA class I type was determined using the LabType SSO (Sequence-specific oligonucleotide probes) typing test (One Lambda) using a Luminex 100 IS (Luminex Corp.) flow analyzer as recommended by the manufacturers. The HEK293T HLA type was confirmed as being homozygous for the haplotype: HLA-A*03; HLA-B*07; HLA-C*07; DRB1*15; DRB5*01; DQB1*06:02; DQA1*01:02, in accordance with the literature [18].

### Cell transfection, cloning and IFN-γ stimulation

Cells were transfected using jetPEI (Polyplus-transfection SA) according to the manufacturer’s protocol in a 24-well cell plate (TPP), and maintained in medium with 100 μg/ml blasticidin (InvivoGen). Cloning was performed by adding ~200 cells to a 150 mm cell culture dish (TPP) to ensure separation of single cells. After 3 weeks, colonies were transferred to a 6-well cell plate (TPP) using cloning filters (Sigma-Aldrich). A share of each of the clones was induced for 72 h with 25 ng/ml IFN-γ (Life Technologies) in a 6-well plate (TPP) and analyzed by direct flow cytometry (see below). Clones were considered positive for the transfected construct if the mean fluorescence intensity exceeded that of the isotype control by 50%, and positive clones were divided into 2 separate wells in a 6-well cell plate (TPP), and either stimulated with 25 ng/ml IFN-γ (Life Technologies) for 72 h or left un-induced, before quantitative flow cytometry (see below).

### Flow cytometry to analyze HLA cell surface expression

Cells were washed twice with phosphate buffered saline pH 7.4 (Life Technologies), and a total of 1 × 10^6^ cells were resuspended in 100 μl of FACS buffer (phosphate buffered saline pH 7.4 containing 2% fetal bovine serum and 2 mM EDTA). The cells were incubated with the primary mouse monoclonal antibody at saturating conditions, as determined by titration, for 30 min at 4°C. For quantitative allele-specific HLA expression measurement, unconjugated anti-HLA-A2 clone BB7.2 (BD Biosciences, Cat. No. 551230) and its isotype control IgG2b clone MPC-11 (BD Biosciences, Cat. No. 557351) were used for the HLA-A2, A2_1-200_/B8_201-362_, A2_1-332_/B8_333-362_ and A2_1-308_/B8_309-362_ constructs, while anti-HLA-B8 biotin-conjugated (OneLambda/ThermoFisher Scientific, Cat. No. BIH0536A) and its isotype control IgG2b (BD Biosciences, Cat. No. 557351) were used for the HLA-B8, B8_1-200_/A2_201-365_, B8_1-332_/A2_333-365_ and B8_1-308_/A2_309-365_ constructs. For expression analyses of HLA-A3 and HLA-B7, unconjugated anti-HLA-A3 clone GAP.A3 (E-Bioscience, custom made) and its isotype control IgG2a (E-Bioscience, Cat. No. 16-4724-82) and anti-HLA-B7 clone BB7.1 (AbD Setotec/Bio-Rad, Cat. No. MCA986) and its isotype control IgG1 (AbD Setotec/Bio-Rad, Cat. No. MCA928) were used, respectively. Screening for positive clones was done by direct immunofluorescence using fluorochrome-conjugated versions of the primary monoclonal antibodies (anti-HLA-A2 PE-conjugated clone BB7.2 (BD Biosciences) and anti-HLA-B8 FITC-conjugated (OneLambda/ThermoFisher Scientific) and the isotype control: IgG2b (OneLambda/ThermoFisher Scientific). Following incubation, the cells were washed twice in FACS buffer and analyzed by direct flow cytometry. For indirect immunofluorescence, the cells were further incubated with the polyclonal FITC-conjugated goat F(ab’)_2_ anti-mouse immunoglobulins secondary antibody (Dako) at saturating conditions for 30 min at 4°C, and washed twice in FACS buffer. Supplier recommended Ab isotype controls coupled with the relevant fluorochrome were used in all the experiments. The cells were analyzed on a CyAn ADP Analyzer (Beckman Coulter). The FACS data were analyzed using SUMMIT 4.3 (Beckman Coulter). For quantitative flow cytometry, the indirect immunofluorescence assay QIFIKIT (Dako) with beads carrying known numbers of murine IgG molecules was used to generate a standard curve according to manufacturer’s protocol.

### RNA isolation and RT-PCR

Total RNA was isolated using the RNeasy Plus mini RNA purification kit (Qiagen) and quantified by NanoDrop spectrophotometry (Thermo Fisher Scientific). The cDNA was synthesized using the RevertAid H Minus first strand cDNA synthesis kit with random hexamer primers (ThermoFisher Scientific).

### Allele-specific quantitative real-time PCR

Quantitative real-time PCR primers were designed and verified according to international guidelines as previously described [14]. The primers used were for HLA-A*02: forward TGAAGGCCCACTCACAGACTC, reverse CCCACGTCGCAGCCATACATC (fragment length 111 bp); for HLA-A*03: forward AAGTGGGAGGCGGCCCATGA, reverse ATGTGTCTTGGGGGGGTCCGT (128 bp); for HLA-B*07: forward CCGTGAGGCGGAGCAGCG, reverse GTCAGCGCGCTCCAGCTTG (99 bp); for HLA-B*08: forward AGAGCCTACCTGGAGGGCAC, reverse CGTGTGTCTTTGGGGGGTCC (97 bp) and for the reference gene FBXL12: forward ACCTGACGCTCTACACGATGC, reverse GAGCCAGAGAACAGGTAGCCA (108 bp). The PCR reaction contained SyBr green PCR master mix (Applied Biosystems), 0.25 mM of each primer and cDNA corresponding to 100 ng of total RNA. Quantitative PCR was performed in a LightCycler 480 from Roche. The reactions were first incubated 2 min at 50°C, and 10 min at 95°C, followed by 40 cycles (15 sec at 95°C, 30 sec at 66°C and 1 min at 72°C) and 10 min at 72°C, and a final DNA dissociation step. For each sample, the cDNA copy number was calculated from a standard curve and divided by the copy number of the reference gene. Standard curves were generated using known numbers of plasmids as templates containing the target sequence in question.

### Statistics

Statistical analyses were performed using GraphPad Prism 4 software. Kruskal-Wallis test was used to test differences between medians for multiple groups. P < 0.05 was considered statistically significant. When significant, the Mann-Whitney U-test was used to test for differences between and chimeric constructs employing Bonferroni correction for multiple comparisons (corrected significance value: 0.05/3 = 0.017). Significant P values are marked with an asterisk next to the uncorrected P value. To test for correlation between cell surface expression and transcript levels, Spearman’s rank order correlation was applied.

## Results

### HEK293T cells have constitutively high cell surface expression of HLA-A but low expression of HLA-B

**Fig 1:**
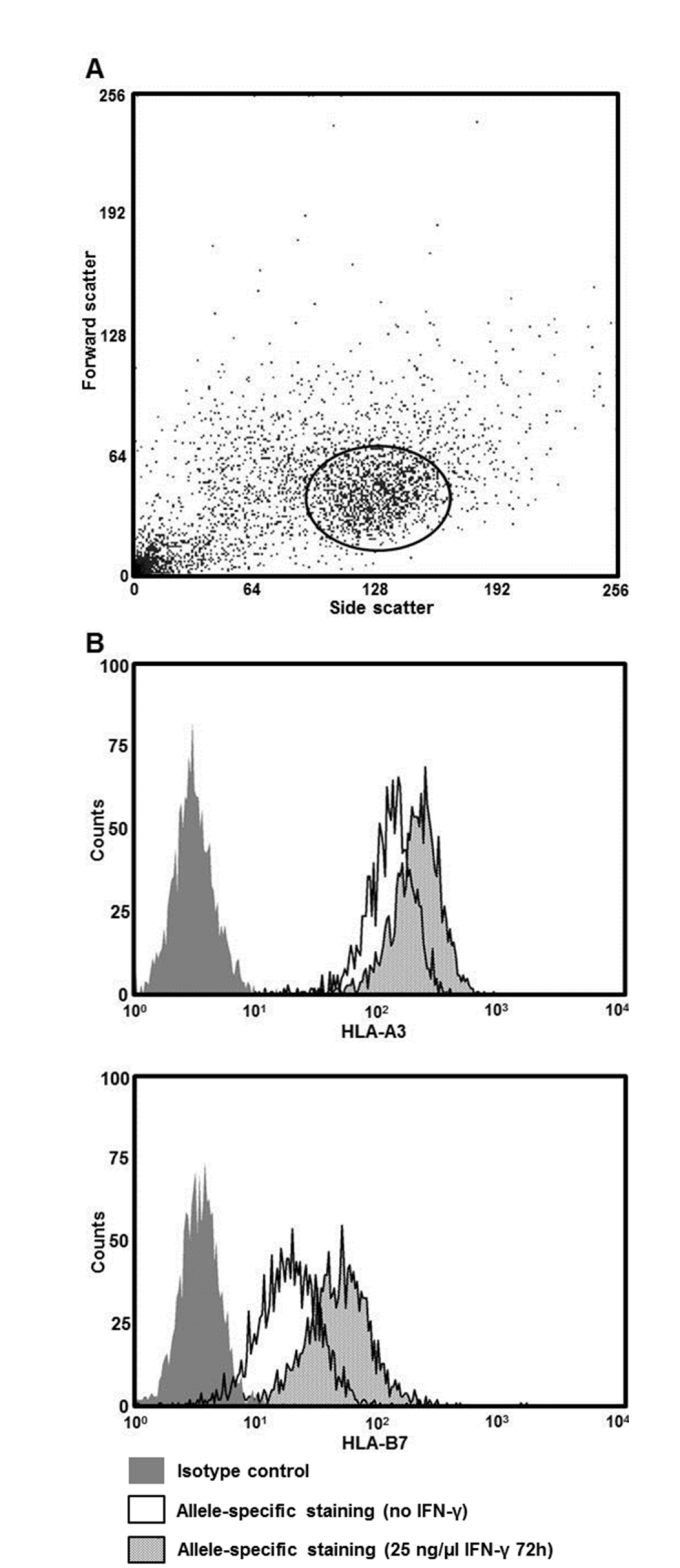
Flow cytometry gating strategy for analyzing HLA cell surface expression on HEK293T cells. Data are representative for all flow cytometry experiments. (A) Forward scatter vs. side scatter dot plot. (B) Histogram showing cell surface expression of HLA-A3 and -B7 using allele-specific antibodies either unstimulated or after IFN-γ stimulation.

**Fig 2:**
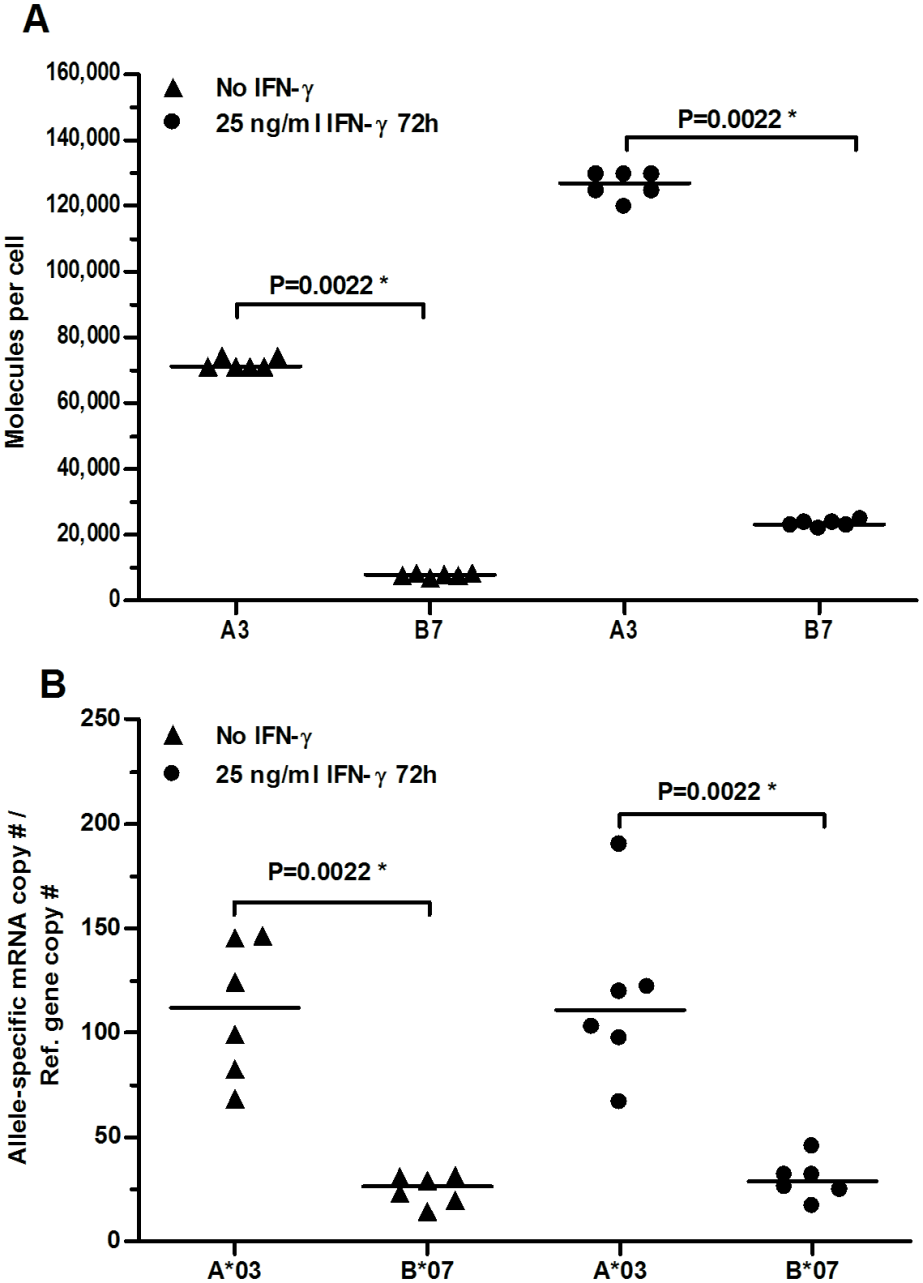
Allele-specific HLA class I cell surface expression and gene transcript levels of HEK293T cells. Horizontal lines indicate median values. (A) Quantitative measurements of HLA class I cell surface expression determined by indirect flow cytometry. (B) HLA class I gene transcripts levels determined by quantitative real-time PCR. * Indicates significant P value (P < 0.05).

The cell surface expressions of HLA-A and -B on HEK293T cells were measured by quantitative flow cytometry using allele-specific primary monoclonal antibodies against either HLA-A3 or -B7, as the cells are homozygous for both (Fig 1). We found that HLA-A3 was highly expressed constitutively with a median of 71,204 molecules per cell (Figs 1B and 2A). That was 9.2 fold higher than that of HLA-B7 (Figs 1B and 2A). Both the expression of HLA-A3 and HLA-B7 increased after stimulation with IFN-γ. HLA-A3 expression was raised 1.8 fold (P = 0.0022) and HLA-B7 3.0 fold (P = 0.0022).

### IFN-γ increases cell surface expression but not mRNA levels of HLA-A and -B in HEK293T

Next, the amount of HLA-A*03 and -B*07 mRNA transcripts were determined using quantitative real-time PCR to investigate whether the difference in expression measured on the cell surface could be a direct consequence of different transcript levels. Indeed, the transcript level of A*03 was 4.3 fold higher than that of B*07. Surprisingly, no significant increase was seen in response to IFN-γ (Fig 2B, A*03: P = 0.94, B*07: P = 0.31) indicating that the increase in cell surface expression was independent of mRNA levels in this cell line and consequently must be regulated by a post-transcriptional mechanism.

### Coding sequence differences between HLA-A and -B have a major effect on cell surface HLA expression

**Fig 3:**
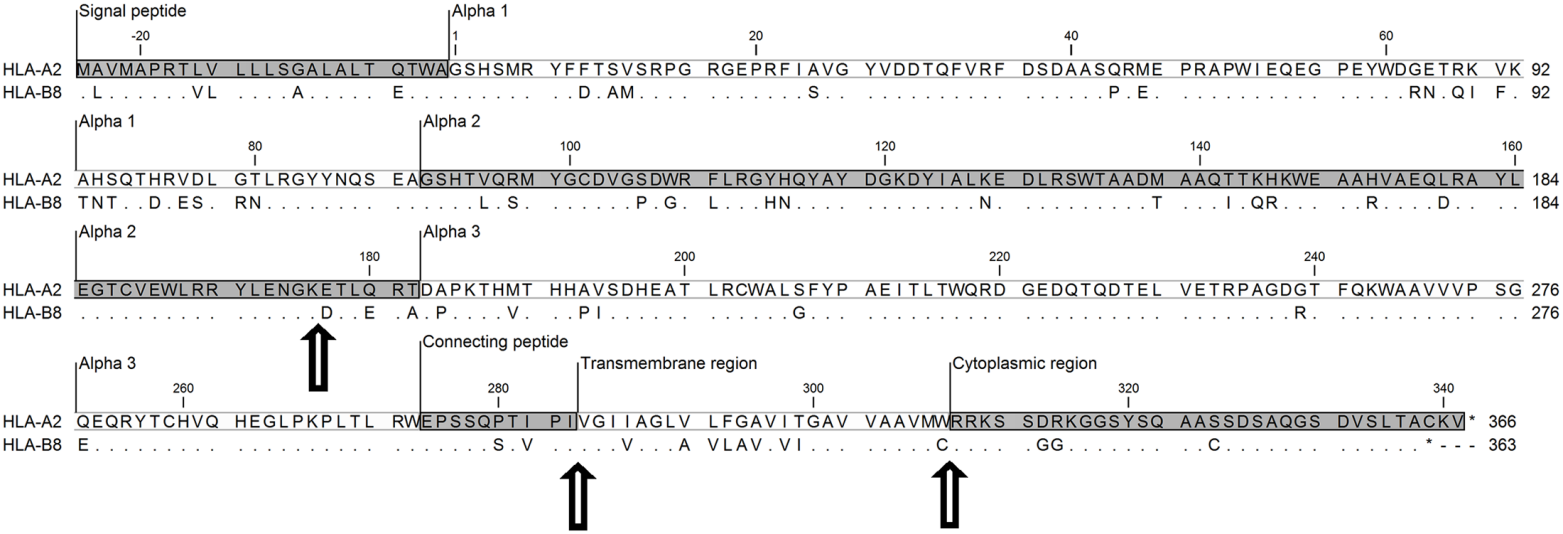
Alignment of amino acid sequences deduced from HLA-A*02:01:01:01 and HLA-B*08:01:01. Identical amino acids are shown as dots. Arrows indicate sites of transition between chimeric constructs. GenBank accession numbers for HLA-A*02:01:01:01, HG794376.1 and for HLA-B*08:01:01, HG794374.1 (http://www.ncbi.nlm.nih.gov/genbank/).

**Fig 4:**
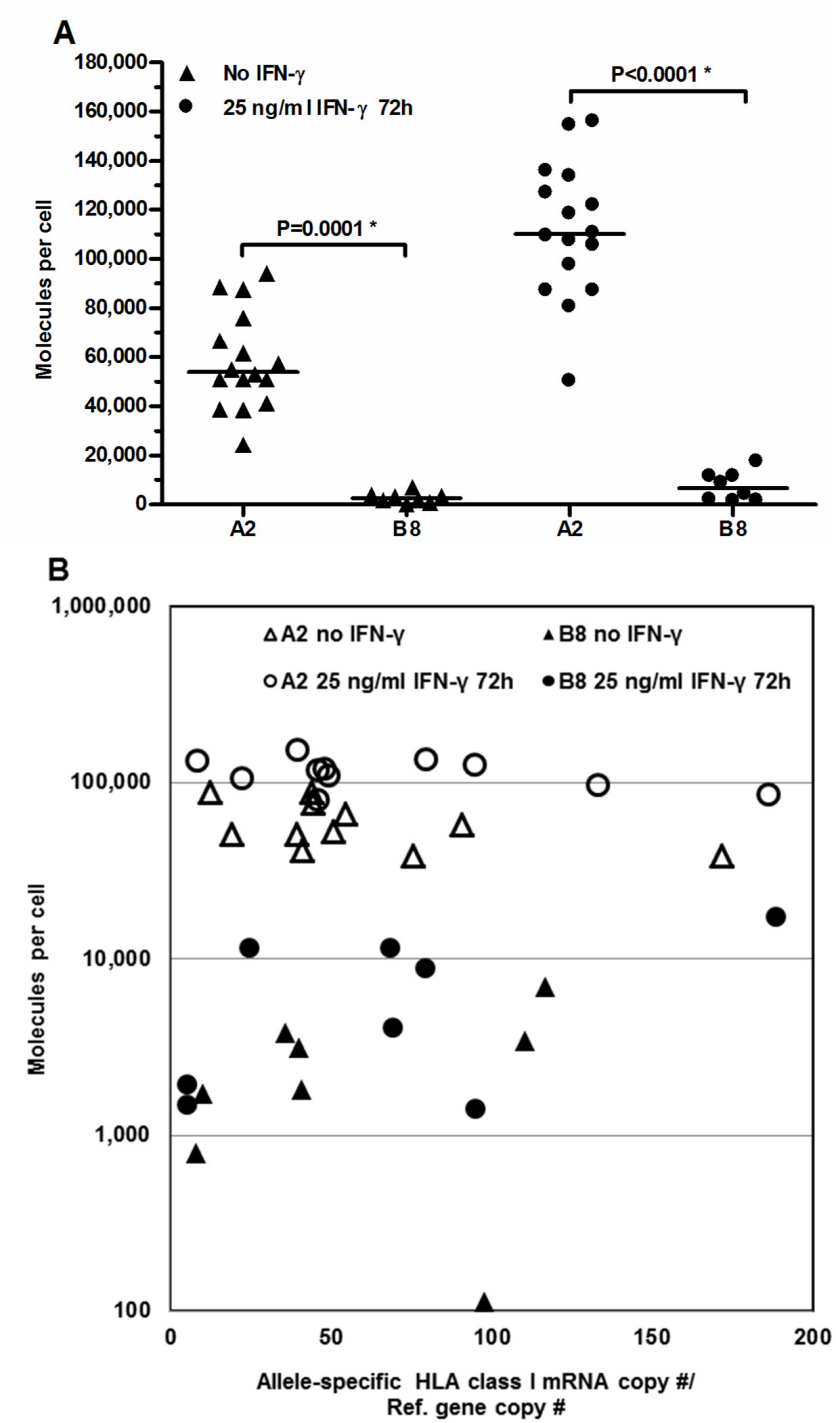
Allele-specific HLA class I cell surface expression and gene transcript levels in cloned HEK293T cells transfected with either A2 or B8 coding sequence constructs. (A) Quantitative measurement performed by indirect flow cytometry of HLA class I cell surface expression. Horizontal lines indicate median values. * Indicates significant P value (P < 0.05). (B) Cell surface protein expression of A2 and B8 as a function of mRNA copy number relative to reference gene copy number.

In order to elucidate the difference in expression between HLA-A and -B, we constructed plasmids containing only the coding sequence of HLA-A*02:01:01 or HLA-B*08:01:01 (Fig 3). These plasmids were separately transfected into HEK293T cells, stably transfected clones were isolated, and the cell surface expression was measured by quantitative flow cytometry using HLA-A2 and -B8 specific monoclonal antibodies (Fig 4A).

The resulting constitutive median expression levels mirrored that of the endogenous molecules when compared locus wise (A2: 54,031 molecules per cell versus 71,204 for A3 in un-transfected cells; 2,466 and 7,778 for B8 and B7, respectively) (Figs 2 and 4A). Thus, the constitutive expression of HLA-B8 was significantly lower than that of HLA-A2 with a 15-fold difference in median expression. When stimulated with IFN-γ, the median expression levels increased 1.9-fold (P<0.0001) for HLA-A2 and similarly but insignificantly 2.7-fold (P = 0.13) for HLA-B8 leading to a 22-fold difference in relative expression level of HLA-A2 and -B8 (Fig 4A).

The gene transcript levels of the HLA-A2 and -B8 transgenic clones were measured during basal un-induced and IFN-γ-induced conditions using HLA allele-specific quantitative real-time PCR, and compared to their cell surface protein expression (Fig 4B). The un-induced mRNA levels were similar for HLA-A*02 and -B*08 (P = 1) and did not change by IFN-γ stimulation (A*02: P = 0.60; B*08: P = 0.73). This was expected because only the coding sequences of HLA-A*02 and -B*08 were present in the vector without their natural regulatory components.

Interestingly, we found no correlation between cell surface protein expression and transcript levels, showing that mRNA availability was not a limiting factor for HLA expression (HLA-A2 no IFN-γ: P = 0.29, IFN-γ stimulated: P = 0.4; HLA-B8 no IFN-γ: P = 0.27, IFN-γ stimulated: P = 0.46). This finding shows that the large variation in cell surface expression of the corresponding HLA-A- and -B molecules was caused by differences in the coding sequences and either resulted from differences in translational speed or in post-translational processing of the proteins.

### The terminal part of the alpha 2 domain and the alpha 3 domain are especially important for cell surface HLA expression

**Fig 5:**
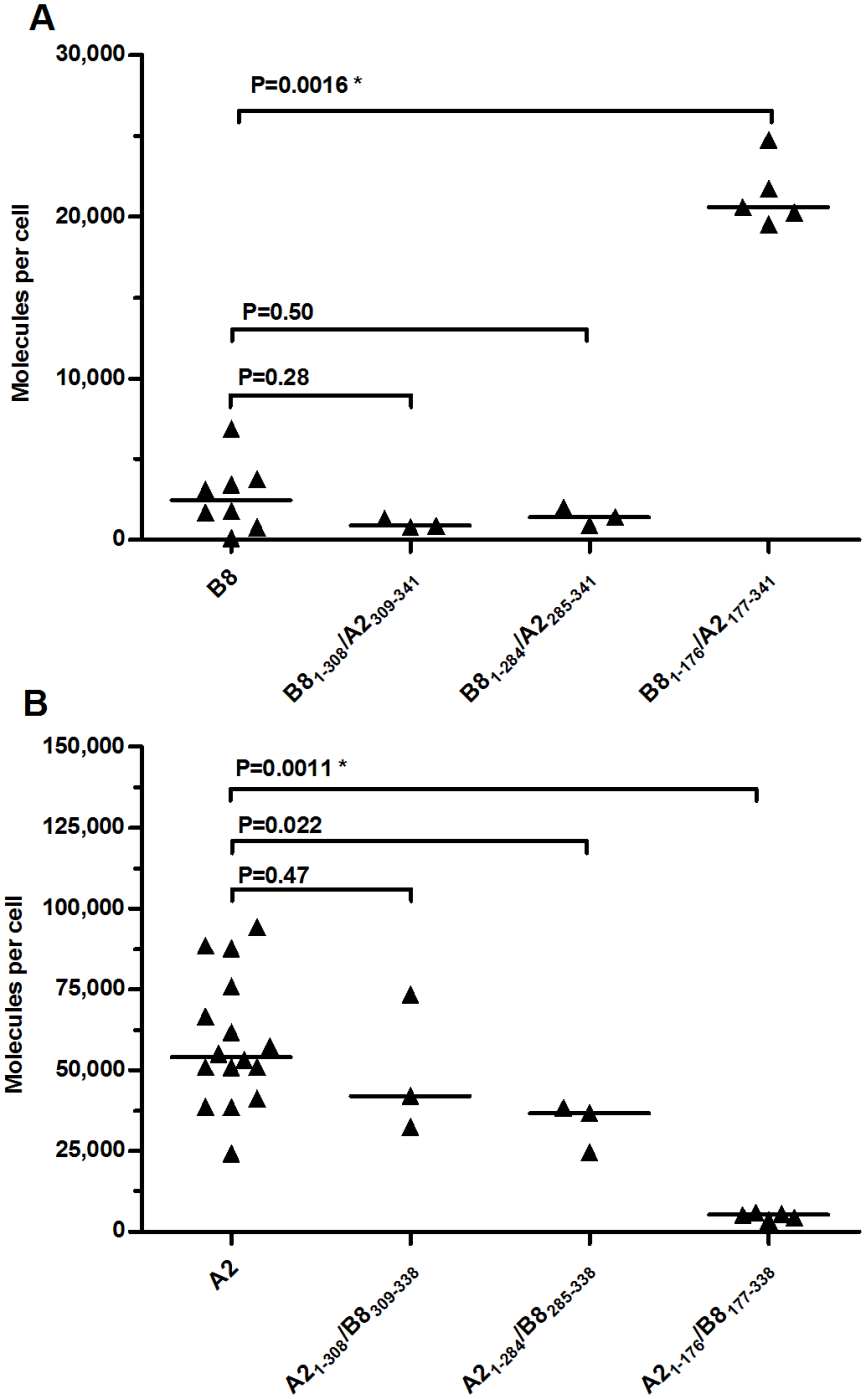
Allele-specific HLA class I cell surface expression levels of cloned HEK293T cells transfected with chimeric constructs. Quantitative measurement determined by indirect flow cytometry, horizontal lines indicate median values. (A) Cells transfected with either B8 or chimeric constructs starting N-terminally as B8 and ending C-terminally as A2. (B) Cells transfected with either A2 or chimeric constructs starting N-terminally as A2 and ending C-terminally as B8. * Indicates significant P value after Bonferroni correction (P < 0.017).

To explore the contribution of the individual domains of the HLA molecule to cell surface HLA expression, several chimeric constructs were made where sequences encoding functional domains of HLA-B8 were replaced with the corresponding domains of HLA-A2 and vice versa (Fig 3). Each of the fusion constructs were transfected into HEK293T cells and chimeric HLA protein expression was measured using anti-HLA-A2 and -B8 specific monoclonal antibodies (Fig 5).

Replacing either the cytoplasmic domain or both the cytoplasmic and transmembrane (TM) domains of HLA-B8 with the corresponding HLA-A2 domains did not have a significant effect on the cell surface expression of the fusion proteins (Fig 5A, constructs HLA-B8_1-308_/A2_309-341_ and -B8_1-284_/A2_285-341_). However, when the exchange between HLA-B8 and -A2 was placed in the terminal part of the alpha 2 domain (construct B8_1-176_/A2_177-341_), the expression of the chimeric construct was 8-fold higher than that of HLA-B8 (Fig 5A). Comparing the expression of the HLA-B8_1-176_/A2_177-341_ and -B8_1-284_/A2_285-341_ chimeric proteins (Fig 5A) with that of HLA-A2 (Fig 5B) indicated that the amino acids 177–284 comprising the six terminal amino acids of the alpha 2 domain and the entire alpha 3 domain (Fig 3) could explain 65% (33,434 molecules per cell) of the absolute difference in cell surface expression of HLA-A2 and -B8 (51,565 molecules per cell).

Similar chimeric constructs were made where HLA-A2 was replaced with HLA-B8 from the terminal part of alpha 2, just before the cytoplasmic region or before the TM region including the cytoplasmic region (Fig 5B). Again, there was no significant effect of replacing the cytoplasmic region or both the TM and cytoplasmic regions (constructs A2_1-308_/B8_309-338_ and A2_1-284_/B8_285-338_). In contrast, when the sequence of the HLA-A2 construct was exchanged with the corresponding sequence of HLA-B8 close to the end of the alpha 2 domain (construct HLA-A2_1-176_/B8_177-338_), it resulted in an 11-fold lower expression (Fig 5B), thus confirming the substantial influence of this part of the molecule.

The cell surface expression levels was also measured after IFN-γ stimulation, where a comparable up regulation between stimulated and unstimulated transfected cells was found for all chimeric constructs (S1 Fig).

### Differences in the coding sequences of HLA-A*02 and -B*08 from the start codon to position 176 in the alpha 2 domain account for a minor part of the difference in cell surface expression

When comparing the expression of HLA-B8 (Fig 5A) with that of the HLA-A2_1-176_/B8_177-338_ chimeric construct (Fig 5B) we found a 2.1 fold (P = 0.045) higher expression of the chimeric construct containing the HLA-A2 sequence. A similar difference was observed when comparing the expression of HLA-A2 to the HLA-B8_1-176_/A2_177-341_ construct (2.6 fold, P = 0.0015). Again, having the HLA-A2 coding sequence resulted in higher expression levels than having HLA-B8 in the same region.

In summary, plasmids encoding HLA-A2 sequences led to much higher cell surface expression than HLA-B8-encoding plasmids, and having HLA-A2 sequences in the terminal part of alpha 2 and in the alpha 3 domain was responsible for most of this effect, while a smaller, but significant effect could be attributed to upstream sequences. In contrast, no consistent effects were seen when only exchanging the TM and/or cytoplasmic regions.

## Discussion

We have previously shown that several human stem cell types and bone marrow-derived T lymphocytes constitutively express HLA-A alleles while only having a very weak or even absent surface expression of HLA-B [14, 15]. For a human mesenchymal stem cell line, we found that this occurred despite the presence of similar levels of mRNA transcripts [14]. This clearly suggested the existence of a hitherto unrecognized allele-specific mechanism of translational or post-translational regulation of MHC class I expression. Such a regulation may have important functional implications because low expression levels of HLA-alleles may interfere with the functions of NK cells and allele-restricted T cells.

In this report, we show that the human cell line HEK293T constitutively exhibits very different expression patterns of HLA-A3 and HLA-B7, the latter being 9.2 times less preponderant on the cell surface, i.e. it expresses a pattern similar to what we analyzed in many normal human stem cell types [14]. Stimulation with IFN-γ increased the cell surface expression of both endogenous and transgenic HLA-A and -B more than two-fold, but retained the ratios between the HLA-A and -B alleles. This stimulation was not associated with an increase in the amount of HLA transcripts and therefore the increased expression had to rely on other IFN-γ-inducible mechanisms like translation, folding, association with B2M, transport, recycling or degradation. In most studied cell types, IFN-γ usually leads to a considerable up-regulation of HLA-transcripts [15] and it was surprising that this was not the case for the endogenous HLA-A*03 and -B*07 alleles in HEK293T. Whereas we have at present no reasonable explanation for this peculiarity of HEK293T, our finding demonstrates that IFN-γ-induced increases in HLA expression are not solely a direct consequence of increased transcription of HLA heavy chain genes.

Using chimeric constructs, we have analyzed the different domains of the HLA-A and -B heavy chains and found that differences in amino acid residues in the terminal part of the alpha 2 and the alpha 3 domains strongly influence cell surface expression levels. In line with this, we found no significant effect of exchanging the cytoplasmic and/or TM domain of HLA-A and -B, and only a minor effect was contributed by the alpha 1 and most of the alpha 2 domains. A valid concern is that exchanging domains between different HLA alleles could disrupt the epitope recognized by the allele-specific monoclonal antibodies. However, the epitope for the HLA-A2 monoclonal antibody used in this study has been mapped and found to be in the alpha 1 and 2 domains (Trp107, Lys127, Gly162 and Arg169) not involving the terminal part of the alpha 2 domain or the alpha 3 domain [19]. The epitope for the HLA-B8 monoclonal antibody has not been determined, but HLA-B7 and HLA-B8 have identical amino acid composition in the alpha-3 domain (HLA-B*07:02:01 GenBank accession number HG794392.1, http://www.ncbi.nlm.nih.gov/genbank/), and because the anti-HLA-B8 monoclonal antibody discriminates between these two tissue types, the epitope must necessarily be situated within the alpha-1 and/or alpha-2 domains. Also, the observation of increased binding of the anti-HLA-B8 monoclonal antibody to cells transfected with the B8_1-176_/A2_177-341_ chimeric construct compared to HLA-B8 transfected HEK293T cells strongly supports the notion that the epitope must be situated in the alpha-1 and/or alpha-2 domains which are the only parts of the B8_1-176_/A2_177-341_ fusion protein derived from HLA-B8. Another concern to be considered is the possibility that the fusion proteins may have intrinsic problems with proper folding. However, this is not likely the case because we, with the exception of a few surface-exposed amino acids in the terminal part of the alpha-2 domain, only exchanged entire functional domains between A2 and B8. Moreover, the observation that all B8-A2 fusion proteins were expressed at least at a level similar to that of the wild type HLA-B8 molecule argues against misfolding. HLA-B8 clearly has no intrinsic difficulty in folding illustrated by the high level of cell surface expression in e.g. B lymphocytes where we have demonstrated an expression level similar to that of HLA-A2 [15].

A study has shown that low-affinity and high-affinity peptide-HLA complexes leave the endoplasmic reticulum (ER) at the same rate, but that peptide-receptive class I molecules are returned from cis-Golgi to the ER [20]. The moderate effect we described, of having HLA-A2-derived amino acids in the alpha 1 and the N-terminal part of the alpha 2 domain compared to having this part of the protein derived from HLA-B8, could result from differences in peptide-binding capacity and consequent susceptibility to peptide loss. Several studies have reported point mutations in the alpha 1 and 2 domains that give rise to differences in expression [21–25]. One study showed that the amino acid in position 114 in the alpha 1 domain is highly correlated with tapasin dependency of peptide loading and thereby cell surface expression. Interestingly HLA-A2 and -B8 differ in this position (H vs. N, Fig 3) [21]. In fact, HLA-A2 is known to be expressed on the cell surface independently of tapasin, while HLA-B8 is partly dependent on its presence [21, 22, 26]. The difference in cell surface expression between HLA-A2 and -B8 in tapasin-deficient cells is, however, much less (approximately 2-fold) than what we observed in HEK293T cells [21]. Moreover, it has recently been shown that HEK-293T cells express tapasin [27] indicating that the lack of this chaperone cannot explain the differences observed in our study. However, other differences in peptide availability (competition between high- or low-affinity peptides) and peptide loading could explain a part of the effect we observed when exchanging the alpha 1 and 2 domains between HLA-A2 and -B8.

The export of MHC class I molecules out of the ER is controversial, as some research groups have reported interactions with cargo receptors [28], while others show that both bulk flow and cargo receptors work in parallel [29]. Bap31 has been shown to associate with HLA-A2, and facilitates the export from the ER [28]. However, the major effect we observed originating from the alpha 3 domain itself makes it less likely that the difference is caused by interactions with the bap31 cargo receptor, as it probably recognizes its ligand in the TM region [30]. While the cytoplasmic domain is of no doubt involved in export from the ER [29], it is not likely causing the observed differential expression of HLA-A2 and -B8, because exchanging the cytoplasmic domains had no effect. Several amino acids in the alpha 3 domain positions 222–229 have been mapped and interacting molecules in the peptide loading complex determined [25, 31–33]. Specifically, it has been shown that HLA-B27 is dependent on the two amino acids Asp227 and Glu229 for interaction with tapasin [25]. These amino acids do not, however, differ between HLA-A2 and -B8 and therefore cannot explain the differential cell surface expression.

In summary, our data indicate that there exists a hitherto unrecognized post-translational difference in the processing of two common HLA molecules within the classical MHC class I Ag-presentation pathway in HEK293T cells. The major effect we evidenced by exchanging the alpha 3 domains between HLA-A2 and -B8, was not caused by differences in already known important amino acids. Whether this difference is caused by different affinity(ies) of HLA-A and -B to a particular component of the peptide loading complex, or to a known or yet undescribed molecule at earlier steps in the HLA processing pathway, remains to be investigated.

## Supporting Information

S1 Fig Effect of IFN-γ on cell surface expression levels of cloned HEK293T cells transfected with chimeric constructs. Quantitative measurement determined by indirect flow cytometry, horizontal lines indicate median values. (A) Cells transfected with either B8 or chimeric constructs starting N-terminally as B8 and ending C-terminally as A2. (B) Cells transfected with either A2 or chimeric constructs starting N-terminally as A2 and ending C-terminally as B8. * Indicates P value significant after Bonferroni correction (P < 0.017).
(TIF)
